# Disseminated Intracranial and Spinal Dysembryoplastic Neuroepithelial Tumor: A Case Report with a Systematic Review

**DOI:** 10.3390/medsci14020250

**Published:** 2026-05-13

**Authors:** Maksymilian Niemczyk, Justyna Fercho, Oskar G. Chasles, Bogdan Jabłoński, Jakub Soboń, Przemysław Nagórka, Maciej Mielczarek, Jacek Szypenbejl, Mariusz Siemiński, Jacek Furtak

**Affiliations:** 1 Scientific Circle of Neurotraumatology, Department of Emergency Medicine, Medical University of Gdańsk, 80-210 Gdańsk, Poland; maksymilian.niemczyk@gumed.edu.pl (M.N.); oskar.chasles@gumed.edu.pl (O.G.C.); bogdan.jabonski@gumed.edu.pl (B.J.); jacek.szypenbejl@gumed.edu.pl (J.S.); mariusz.sieminski@gumed.edu.pl (M.S.); 2 Neurosurgery Department, 10th Military Research Hospital and PolyClinic SPZOZ in Bydgoszcz, 85-681 Bydgoszcz, Poland; drsobon@gmail.com (J.S.); pnagorka@wp.pl (P.N.); jacek.furtak2019@gmail.com (J.F.); 3 Department of Emergency Medicine, Medical University of Gdańsk, 80-210 Gdańsk, Poland; 4 Lung Transplant Unit, Cardiac Surgery Department, Medical University of Gdańsk, 80-210 Gdańsk, Poland; 5 Neurosurgery Department, Stanisław Staszic Specialist Hospital, 64-920 Piła, Poland; maciejmielczarek87@gmail.com; 6 Faculty of Medicine, Bydgoszcz University of Science and Technology, 85-796 Bydgoszcz, Poland

**Keywords:** dysembryoplastic neuroepithelial tumor, DNET, multifocal DNET, spinal DNET, intracranial DNET, concurrent DNET

## Abstract

**Background:** Disseminated intracranial and spinal dysembryoplastic neuroepithelial tumors (DNETs) are exceptionally rare, with only five prior cases reported. **Methods:** This study presents a case of a 47-year-old woman with DNETs in the right hippocampus and lumbar spine, treated surgically, and systematically reviews the existing literature (total *n* = 6). **Results:** While primary tumors occurred in both locations, secondary lesions were predominantly spinal (83%). The key features included frequent obstructive hydrocephalus (67%, often requiring shunting) but rare epilepsy (*n* = 1, 17%). Two fatal outcomes were possibly associated with West Nile virus infection (the S100B protein pathway). MRI was performed in all cases, and surgical intervention (craniotomy/laminectomy) constituted the primary treatment modality (67%). **Conclusions:** This multifocal presentation poses significant challenges, underscoring the potential need for spinal MRI in intracranial DNET cases and multidisciplinary management. Further molecular studies and case registries are crucial to understand pathogenesis and optimize care.

## 1. Introduction

Dysembryoplastic neuroepithelial tumors (DNETs) are benign, glioneuronal neoplasms classified as WHO grade 1, predominantly affecting the cerebral cortex [1]. First described in 1988, DNETs are characterized by a heterogeneous composition of neuronal and glial elements, often presenting as cortically based lesions with a multinodular architecture [2]. These tumors are strongly associated with medically refractory epilepsy, particularly in children and young adults, with a peak incidence in the second decade of life [3]. Their benign nature and low proliferative potential typically result in favorable outcomes following surgical resection, which is the primary treatment modality [4]. Neuroimaging, particularly magnetic resonance imaging (MRI), reveals well-demarcated, T2-hyperintense lesions, often in the temporal or frontal lobes, with minimal mass effect or contrast enhancement [5]. Histologically, DNETs are distinguished by a specific glioneuronal element, including oligodendrocyte-like cells and floating neurons within a mucoid matrix [1].

While cerebral DNETs are relatively well-documented, with an estimated prevalence of 0.6–0.7% among pediatric brain tumors [6], their occurrence in extracerebral locations, such as the spinal cord, is exceptionally rare. Spinal DNETs are sparsely reported, with 5 cases in the literature, typically presenting with symptoms like radiculopathy or myelopathy due to cord compression. The rarity of spinal involvement is attributed to the embryologic origins of DNETs, which are thought to arise from secondary germinal layers during cortical development, a process less relevant to spinal cord ontogeny. Notably, all documented cases of DNETs occurring in the spinal cord were ultimately diagnosed as a dual manifestation, presenting in both the spinal cord and cerebrum or infratentorially. A comprehensive literature review identified only five such cases, highlighting an exceedingly uncommon multifocal presentation and pointing out that isolated spinal DNET lesions are yet to be described. These dual-location cases pose unique diagnostic and therapeutic challenges, as the spinal component may be mistaken for other pathologies, such as ependymomas or astrocytomas, on imaging. This case represents a disseminated intracranial and spinal dysembryoplastic neuroepithelial tumor (DNET) since it documents the sixth reported instance worldwide of such a multifocal involvement (with only five prior cases identified in the systematic review for the period 2009–2024), with our patient being the oldest described at the age of 47, featuring a hippocampal primary lesion with uncal herniation alongside a distinct lumbar spinal secondary lesion confirmed histopathologically for the intracranial component and surgically managed for both sites, achieving seizure freedom post-resection. Furthermore, the case is distinctive due to the rare occurrence of false lateralization on EEG (epileptiform activity predominantly contralateral to the hippocampal lesion), the discordant imaging enhancement patterns between the non-enhancing intracranial and enhancing spinal components, and the absence of epilepsy as a dominant feature in most multifocal DNETs (present in only 17% of reviewed cases), highlighting atypical clinical behavior compared to typical solitary cortical DNETs.

## 2. Case Report

A 47-year-old woman was referred to a neurosurgical department, reporting dizziness, a heavy head sensation, and focal epilepsy characterized by a warm sensation in the upper abdomen every 10 days, lasting approximately 12 s, followed by dizziness. She also experienced episodic nocturnal anxiety but denied memory loss or olfactory disturbances.

Physical examination revealed no neurological deficits (no ataxia and a negative Romberg test) with a Karnofsky Performance Status (KPS) score of 90.

**Figure 1:**
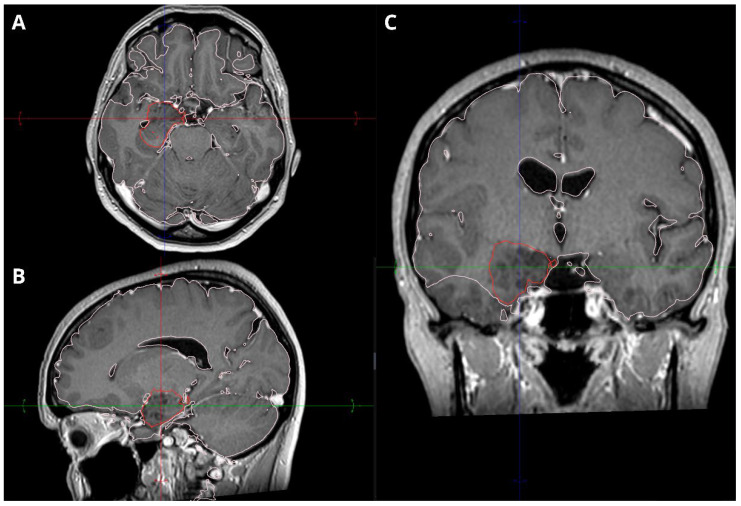
Primary dysembryoplastic neuroepithelial tumor (DNET) in the right hippocampus of a 47-year-old female at presentation. (**A**) Axial post-contrast T1-weighted MRI showing a well-demarcated, heterogeneous lesion in the right medial temporal lobe, with uncal herniation. (**B**) Sagittal post-contrast T1-weighted MRI revealing a medially located mass without significant brainstem deformation. (**C**) Coronal post-contrast T1-weighted MRI demonstrating the mass in contact with the ventricular wall, with no evidence of midline shift.

**Figure 2:**
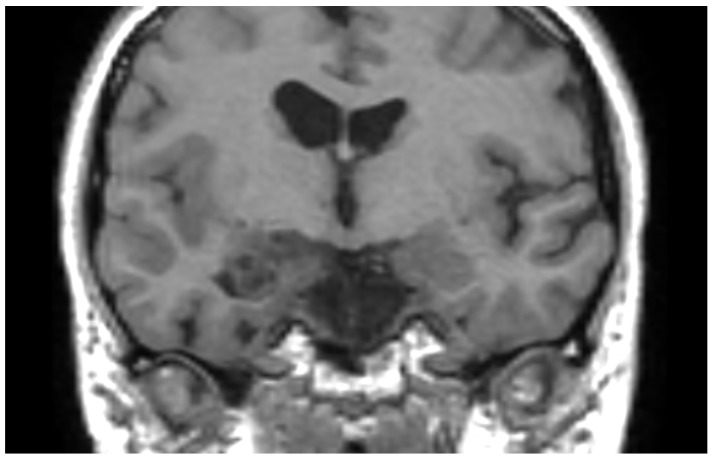
Coronal non-contrast T1-weighted MRI of the patient’s right hippocampus DNET at presentation. There is no evidence of the medially located lesion provoking midline shift or surrounding edema.

**Figure 3:**
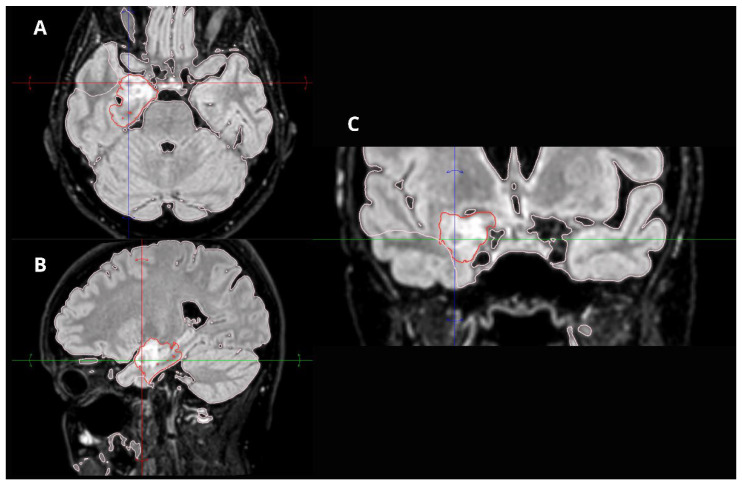
Preoperative FLAIR MRI of the patient’s dysembryoplastic neuroepithelial tumor (DNET) in the right temporal lobe. (**A**) Axial FLAIR scan showing a clearly delineated lesion with central hypointensity, suggestive of possible necrosis. (**B**) Sagittal FLAIR scan depicting the lesion’s extent and its well-defined borders. (**C**) Coronal FLAIR scan highlighting the lesion’s relationship to surrounding structures, with central hypointensity consistent with potential necrosis.

**Figure 4:**
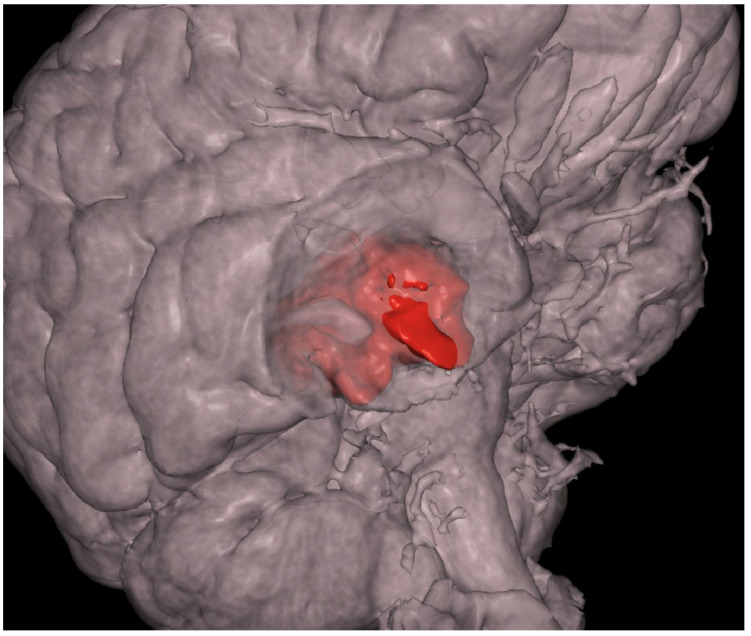
The 47-year-old patient’s preoperative 3D reconstruction of the dysembryoplastic neuroepithelial tumor (DNET) in the right temporal lobe. The lesion is highlighted in red for visual reference, illustrating its volume and spatial relationship to surrounding structures.

**Figure 5:**
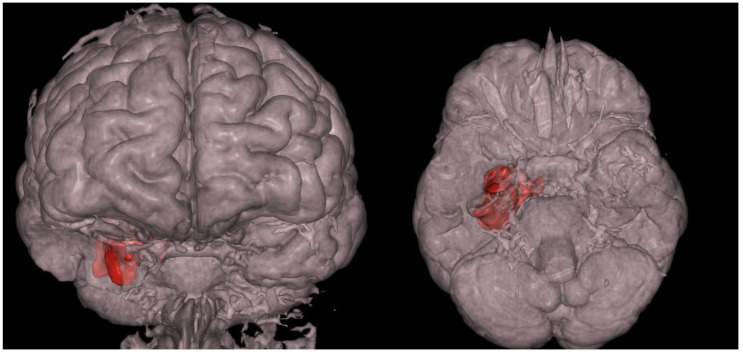
The 47-year-old patient’s preoperative 3D reconstruction in the anterior and inferior view respectively. Uncal herniation of the DNET lesion is more clearly presented in these illustrations.

MRI revealed a non-enhancing right hippocampal lesion, initially suspected to be a low-grade glioma (LGG) or dysplastic lesion. The mass showed uncal herniation (Figure 1, Figure 2, Figure 3, Figure 4 and Figure 5).

EEG showed epileptiform abnormalities in the frontal and frontotemporal leads, predominantly on the left. We interpret it as a case of false lateralization, which is the most common for temporal lesions, including the hippocampus. It is primarily related to temporal cavernomas [7].

**Figure 6:**
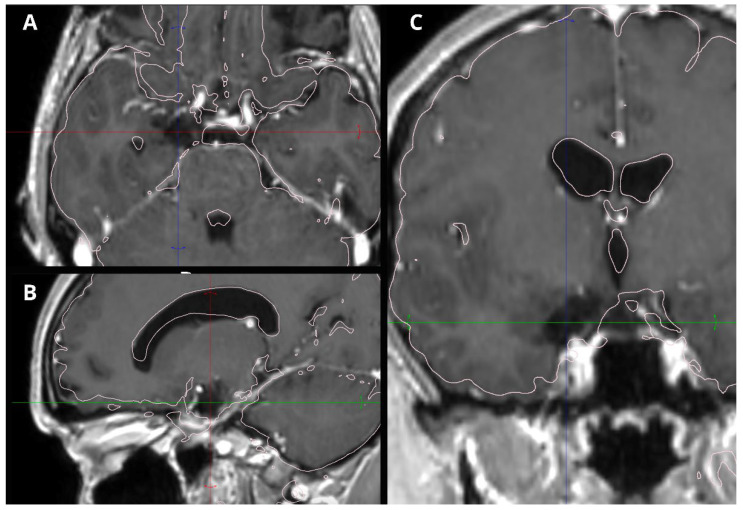
Post-contrast T1-weighted MRI following gross total resection (GTR) of a dysembryoplastic neuroepithelial tumor (DNET) in the right hippocampus via supraorbital craniotomy. (**A**) Axial scan showing the resection cavity with no residual tumor. (**B**) Sagittal scan confirming complete tumor removal and preservation of adjacent structures. (**C**) Coronal scan demonstrating the absence of residual lesion and normal alignment of surrounding anatomy.

**Figure 7:**
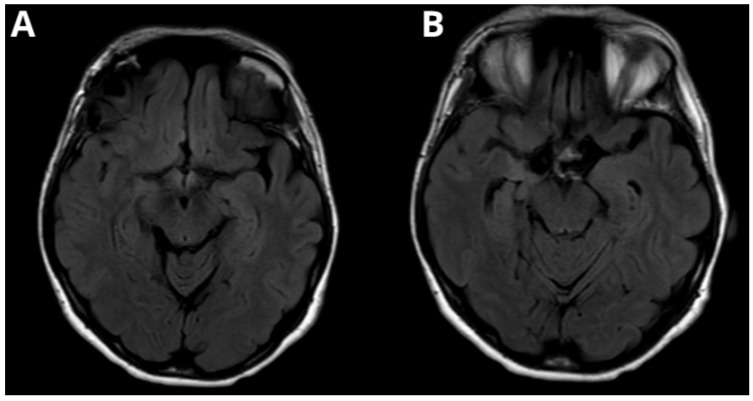
Postoperative FLAIR MRI of the patient’s dysembryoplastic neuroepithelial tumor (DNET) in the right temporal lobe. (**A**) Axial scan showing no residual tumor and stability of intracranial anatomical structures following surgical intervention. (**B**) Axial scan at a more caudal level to scan B, confirming complete resection and showing the resection cavity.

A right-sided supraorbital craniotomy aiming for lesionectomy achieved gross total resection (GTR) of the hippocampal lesion, confirmed by postoperative CT and MRI (Figure 6 and Figure 7). This surgical approach was chosen due to its minimal invasiveness and suitability for medial temporal lesions. Postoperatively, due to the lesion’s infiltration of the nerve, the patient developed transient right oculomotor nerve paresis, which improved during hospitalization. Histopathological examination confirmed the lesion as a dysembryoplastic neuroepithelial tumor (DNET). The patient remained under follow-up, systemically coming back for checkups in our clinic, to monitor the symptoms. She has remained seizure-free since the surgery.

Five months post-discharge, the patient returned to the clinic reporting lumbosacral pain and was referred for a routine follow-up MRI of the brain and, additionally, the lumbar spine in light of the newly emerged symptom.

**Figure 8:**
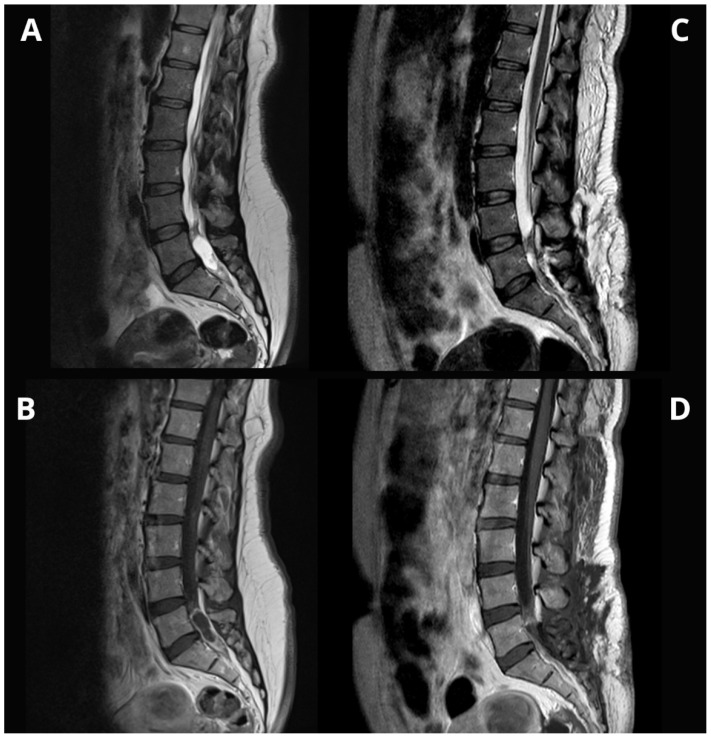
Pre- and postoperative imaging comparison of a dysembryoplastic neuroepithelial tumor (DNET) disseminated lesion in the lumbosacral region. (**A**) Preoperative T1-weighted MRI showing the DNET secondary lesion at the mid-L5 level, extending downward into the sacral canal with peripheral enhancement. (**B**) Postoperative T1-weighted MRI following L5 laminectomy, demonstrating complete resection of the spinal lesion with no residual tumor. (**C**) Preoperative STIR MRI highlighting the lesion’s extent and peripheral enhancement. (**D**) Postoperative STIR MRI confirming successful resection with no evidence of residual lesion or abnormal signal intensity.

MRI (FLAIR and DWI) of the head showed no recurrence. However, a lumbar-sacral (L-S) MRI revealed an unclear lesion in the lumbar cistern from mid-L5 to the sacral canal, without significant stenosis. A follow-up MRI 2 months later identified an intradural L4 lesion, consisting of a peripherally enhancing cyst and a solid tumor with homogeneous post-contrast enhancement, extending to the sacral canal and compressing spinal nerves. The L5-S1 lesion was evaluated preoperatively using STIR sequence imaging and resected via L5 laminectomy with intraoperative neuromonitoring. (Figure 8).

One month later, the patient presented to the emergency department with a 2-day history of lumbosacral pain radiating to the left lower limb through the buttock to the lateral malleolus, without paresis or sensory deficits. The lumbosacral pain was secondary to the loosening of a previous laminoplasty construct, which necessitated a removal in a subsequent procedure. The pain was managed with thermal percutaneous facet joint denervation at L3/L4 and L4/L5, resulting in symptom resolution. Histopathological examination determined the spinal lesion to be a disseminated DNET.

The decision to forego adjuvant treatment after both resections was reached by a multidisciplinary team consensus. Despite the omission of adjuvant radiochemotherapy, the patient maintains a stable, recurrence-free status at the latest follow-up.

As of April 2026, the patient continues to be followed up in an outpatient clinic every 6 months. The patient has now been followed up for 20 months, and the decision regarding the patient’s eventual conclusion will be made on a continuous basis during future reassessments.

We received the patient`s informed consent to publish this case report and any accompanying images.

## 3. Methods of Literature Review

A systematic review was conducted by the research team, analyzing a dataset of six case reports, comprising five published studies (2009–2024) identified through a comprehensive literature search (M.N.; O.C.) and the current case of a 47-year-old woman with both intracranial and spinal dysembryoplastic neuroepithelial tumor (DNET). Two independent reviewers screened titles and abstracts of retrieved records, followed by full-text assessment of potentially eligible studies. A comprehensive literature search was performed on PubMed, Embase, Scopus and Web of Science databases.

The search strategy was: (“dysembryoplastic neuroepithelial tumor” OR “DNET” OR “DNT”) AND (“intracranial” OR “spinal” OR “concurrent” OR “multifocal” OR “disseminated”). No additional filters were applied.

Studies were excluded if they lacked sufficient clinical, radiographic, or histopathological details or reported only solitary intracranial or spinal DNETs without multifocal involvement or if there were any other central nervous system neoplasms involved concurrently.

Due to the qualitative nature of the included case reports, a formal risk of bias assessment was not conducted. However, the quality of reporting was evaluated based on the completeness of clinical, radiographic, and histopathological information provided in each study.

Variables collected included sex, age at initial diagnosis, initial diagnosis, primary tumor localization, metastasis location, fatal outcome, molecular genetic alterations, obstructive hydrocephalus, epilepsy, treatment for hydrocephalus, primary symptoms, postoperative symptoms, imaging modalities (CT non-contrast, CT contrast, MRI non-contrast, MRI contrast, FLAIR, PET, FIESTA, DWI, STIR, EEG), surgery performed (other than shunts), treatment, biopsies performed, immunohistochemistry, follow-up, West Nile Virus (WNV) occurrence, cerebrospinal fluid (CSF) findings, and other relevant notes. Descriptive statistics, including frequencies, percentages, means, and ranges, were calculated to summarize patient characteristics, clinical features, and outcomes. Missing data were documented, and no inferential statistical tests were conducted due to the small sample size and qualitative nature of the case reports.

**Figure 9:**
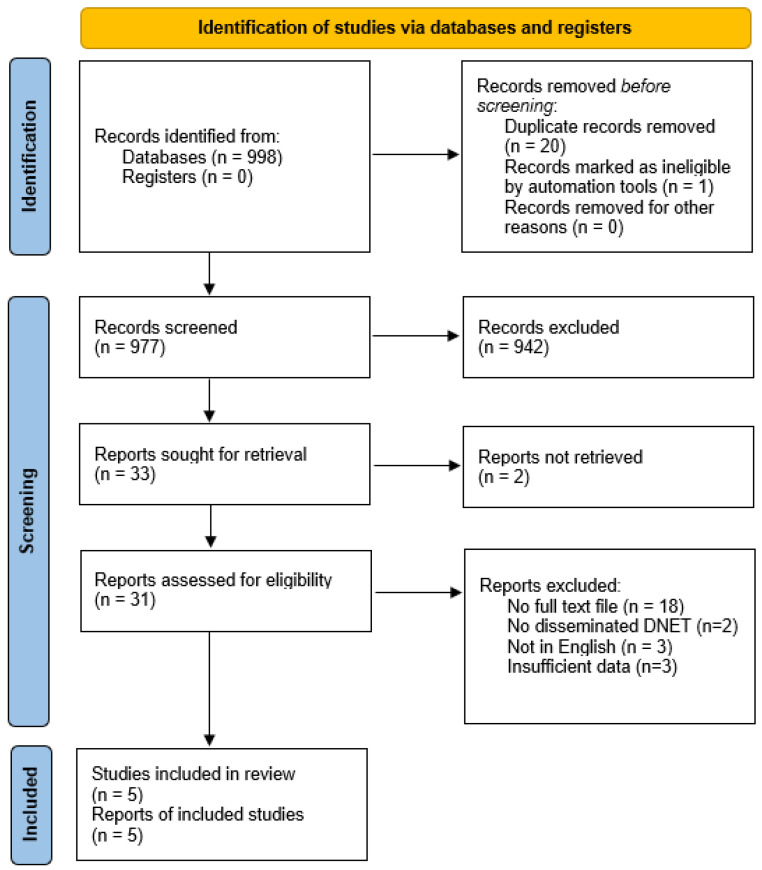
PRISMA Flowchart.

The results of individual studies were tabulated in a summary table (Table 1) and primary symptom data were visualized in a chart (Figure 9) to depict their distribution and frequency. A narrative synthesis was performed due to the small sample size and heterogeneity of case reports, with descriptive statistics used to summarize findings. No meta-analysis was conducted.

This systematic review was registered and accepted in the PROSPERO database. (registration number: CRD420251052762).

Graphics based on MRI scans were processed and prepared using Inobitec DICOM Viewer, which enabled a detailed spatial representation of the dysembryoplastic neuroepithelial tumor (including 3D reconstruction of the intracranial lesion).

This research received no external funding or financial support.

The manuscript adheres to PRISMA 2020 guidelines (Figure 9).

## 4. Results

We analyzed six case reports (2009–2024), including the one described by our group above. The research team performed data extraction and analysis without artificial intelligence tools. The data was compared and presented in Table 1. Variables included patient demographics, tumor characteristics, clinical features, imaging, treatments, and outcomes. Descriptive statistics summarized findings, with missing data noted. No inferential tests were conducted due to the small sample size and qualitative nature of the case reports.

**Figure 10:**
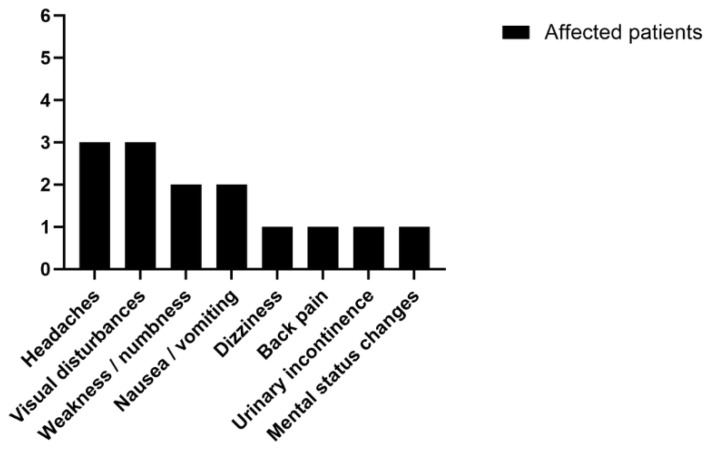
The distribution of presenting primary symptoms in patients diagnosed with intracranial and spinal DNETs.

Primary symptom data were visualized using a chart, as shown in Figure 10, to depict their distribution and frequency. Other patient-related data, including tumor characteristics, were not graphically represented, as the small sample size (*n* = 6) was deemed insufficient to allow for meaningful interpretation.

Among the six patients, three (50%) were female, two (33.3%) were male, and one case (16.7%) had unspecified sex. The mean age at diagnosis was 29.7 years (range: 9–47), with two patients (33.3%) classified as children/adolescents (≤22 years) and four (66.7%) as adults. Initial diagnoses varied: one case each (16.7%) of intraventricular astrocytoma, differentiation between oligodendroglioma/central neurocytoma/subependymal giant cell astrocytoma/DNET, leptomeningeal spread from a thoracic lesion, unknown third ventricle lesions, prior West Nile Virus (WNV) effect, and histologically confirmed DNET (Case 6). Primary tumors were located in the ventricles (*n* = 3, 50%), right hippocampus with uncal herniation (*n* = 1, 16.7%), thoracic (*n* = 1, 16.7%), or lumbar spinal cord (*n* = 1, 16.7%). Secondary lesions were predominantly spinal (*n* = 5, 83.3%), affecting cauda equina, cervicothoracic, filum terminale, or lumbar/sacral regions, with intracranial involvement (cerebellum, brainstem, frontal/temporal lobes) in two (50%). Two cases (33.3%), both WNV-associated, were fatal. Molecular alterations were reported in one case (16.7%, FGFR1p.N546K mutation, no BRAF V600E).

Obstructive hydrocephalus occurred in four cases (66.7%), treated with ventriculoperitoneal shunts (two cases), third ventriculostomy (one case), or septostomy/shunt (one case). Epilepsy was reported only in Case 6 (16.7%). Common symptoms included headaches and visual disturbances (*n* = 3, 50% each), with case-specific complaints like dizziness (Case 6) or urinary incontinence (Case 3). Postoperative symptoms included pain and weakness (*n* = 2, 33.3% each), with outcomes like seizure freedom (Case 6) or quadriplegia (Case 5).

All cases used non-contrast and contrast-enhanced MRI, with FLAIR in four (66.7%). Non-contrast CT (*n* = 2, 33.3%), FIESTA, DWI, and STIR (*n* = 1, 16.7% each) were less common, and PET was unused. EEG, showing epileptiform abnormalities, was used in Case 6. Surgical interventions included craniotomy/laminectomy (Case 6), transcallosal approach (Case 2), laminectomy (Case 3), or biopsy (Case 4), with two cases (33.3%) having no surgery. Treatments included radiation (n = 2, 33.3%), pain management (*n* = 1, 16.7%), steroids/hospice (*n* = 1, 16.7%), or no radio-/chemotherapy (*n* = 1, 16.7%). Biopsies (*n* = 4, 66.7%) showed synaptophysin or no GFAP in one case each. Follow-up ranged from 3 to 4 months (worsening, Case 3) to 1–3 years (stable, Case 2), with Case 6 seizure-free.

Notably, case 2 showed differential contrast enhancement (none intraventricular, present spinal), case 3 had meningeal carcinomatosis, Case 4 received radiotherapy without endocrine issues, and case 6 had no spinal stenosis. WNV IgM antibodies were detected in the two fatal cases’ of cerebrospinal fluid.

In summary, primary tumors were evenly split between ventricles and other sites, with spinal secondary lesions predominant.

Hydrocephalus was frequent, epilepsy was rare, and outcomes varied from seizure freedom to fatal progression, particularly with WNV.

## 5. Discussion

Dysembryoplastic neuroepithelial tumors (DNETs) are rare, benign World Health Organization (WHO) grade 1 glioneuronal neoplasms that typically present as cortically based lesions in the temporal lobe and are strongly associated with refractory epilepsy in children and young adults [1]. This report of a 47-year-old woman with a histologically confirmed right hippocampal DNET protruding to skull base cisterns and a lumbar spinal lesion represents an exceptionally rare multifocal presentation. Our systematic review of five additional cases (2009–2024) underscores the scarcity and complexity of disseminated intracranial and spinal tumors, with only the index case definitively diagnosed as DNET [8,9,10,11,12].

In the index case, focal epilepsy characterized by abdominal warmth and dizziness, coupled with non-enhancing MRI findings, aligned with classic DNET features [5]. In contrast, the spinal lesion presented with radicular pain and homogeneous contrast enhancement, suggesting a distinct radiographic profile potentially reflective of histopathological or microenvironmental differences [3,11]. Notably, epilepsy was reported in only 17% of multifocal cases, a stark contrast to the high epileptogenicity of typical cortical DNETs, suggesting that multifocal presentations may lack the epileptogenic hallmark of solitary lesions [4]. The predominance of symptoms such as headaches (50%) and visual disturbances (50%) in reviewed cases highlights the mass effect of ventricular or spinal lesions, with hydrocephalus occurring in 67% of patients, often necessitating ventriculoperitoneal shunts [8,10,11].

Supporting the consensus that surgical intervention remains the cornerstone of treatment for symptomatic DNETs—having been performed in 67% of the reviewed cases—our index case achieved highly favorable outcomes through a comprehensive surgical strategy. For the intracranial component, a gross total resection (GTR) of the hippocampal lesion via a supraorbital craniotomy successfully achieved complete seizure freedom. This outcome strongly aligns with established literature identifying GTR as a primary prognostic factor for optimal seizure control [2,4]. While this cranial approach was complicated by a transient postoperative oculomotor nerve paresis, the deficit fully resolved, underscoring the critical need for meticulous preoperative planning when navigating eloquent regions [2]. In retrospect, this complication was potentially preventable and appears to have been secondary to the selected surgical corridor. A subtemporal approach might have been a more effective alternative for minimizing the risk of such a complication. Concurrently, the spinal lesion was addressed via an L5 laminectomy, which effectively alleviated the patient’s compressive symptoms. The risk of laminoplasty failure, characterized by construct loosening and later removal, warrants thorough consideration during the preoperative planning for spinal DNET dissemination. Although postoperative radicular pain subsequently necessitated facet joint denervation, the success of this adjunctive intervention highlights the importance of a stepwise, multidisciplinary approach to managing residual symptoms. Ultimately, the positive trajectory of our index case, along with one other in the literature, reinforces the efficacy of well-planned surgical resection coupled with targeted symptom management.

Our literature review revealed a wide anatomical distribution of primary DNETs—spanning the ventricles, hippocampus, and spinal cord—with secondary lesions presenting predominantly in the spine (83%), particularly along the cervicothoracic region, cauda equina, and sacral nerve roots. Although the exact mechanisms of this spread remain poorly understood, this distribution strongly suggests a natural propensity for spinal dissemination. Interestingly, the factors driving this aggressive progression may occasionally involve external, infectious triggers. For instance, two fatal cases were possibly associated with West Nile Virus (WNV) infection, as confirmed by IgM antibodies in the cerebrospinal fluid [10,12]. While a direct causal link in DNETs remains speculative, experimental evidence indicates that WNV could potentially exacerbate tumor aggressiveness and promote gliomagenesis via S100B protein pathways [13]. Beyond these potential viral influences, our broader understanding of multifocal DNET pathogenesis is severely hampered by a scarcity of molecular data. With only a single reviewed case reporting an *FGFR1* p.N546K mutation, there remains a critical gap in the literature regarding the genetic underpinnings of these complex tumors, underscoring the need for comprehensive molecular profiling in future studies [11]. FGFR alterations are increasingly recognized as driver mutations in low-grade neuroepithelial tumors (LGNT), considered as a potential target for inhibition therapy (which still remains an area of investigation) [14]. With DNETs being among those tumors often harboring those mutations, the role of FGFR inhibitors may be an effective alternative in tumors resistant to conventional anti-seizure treatment [14,15].

Diagnosing multifocal DNETs is challenging, as spinal lesions are frequently mistaken for astrocytomas or ependymomas due to their contrast-enhancing patterns, unlike the typically non-enhancing intracranial DNETs [3]. In the index case, the spinal lesion’s enhancement contrasted with the non-enhancing hippocampal lesion, complicating differentiation between multifocality and metastasis [6]. All cases relied on non-contrast and contrast-enhanced MRI, with FLAIR imaging used in 67%, but advanced modalities like diffusion-weighted imaging (DWI) or positron emission tomography (PET) were underutilized, indicating a dependence on conventional imaging [5]. The identification of a lumbosacral DNET may warrant whole-spine imaging to screen for potential dissemination; however, this was not performed in the present case due to financial constraints. The decision to omit MRI of the remaining spinal segments was also supported by the fact that the patient’s symptoms were strictly localized to the lumbosacral region.

The findings presented in our case strongly suggest the potential for tumor spread via cerebrospinal fluid (CSF) pathways. Such an observation challenges the conventional understanding of DNET clinical behavior and indicates that they may occasionally exhibit a more aggressive, infiltrative course. Despite the potential for CSF-mediated spread, cytological evaluation was not performed in our patient, representing a diagnostic gap in our follow-up protocol. We acknowledge that in such cases, proactive CSF screening should be prioritized—similar to ependymoma management—to allow for the timely consideration of adjuvant therapeutic interventions.

Consistent with the generally benign nature of DNETs—which typically precludes the use of chemotherapy—surgical resection served as the primary treatment in 67% of reviewed cases, while radiation therapy was reserved for only two instances involving residual or disseminated disease. Interestingly, the clinical management of multifocal cases often diverges from that of typical DNETs. Because these patients present with a lower incidence of epilepsy, routine antiepileptic therapy becomes less central to their care, shifting the therapeutic focus toward mitigating mass effects and alleviating localized pain [4]. This evolving paradigm is perfectly illustrated by our index case, wherein targeted denervation successfully achieved symptom control. Ultimately, because long-term outcomes vary drastically—ranging from complete seizure freedom and stable disease to fatal progression in rare WNV-associated instances—it is clear that treatment plans must be highly individualized to address the specific anatomical and symptomatic challenges of each patient [13].

The unexpected mortality seen in disseminated DNETs leads us to believe radiosurgery might be considered a viable option for salvage therapy. Salvage gamma knife radiosurgery has been shown to display promising outcomes in intracranial DNETs [16] and fractionated radiosurgery is mentioned as a valuable alternative to single-stage treatment [17] and the use of radioenhancers promise to reduce the radiation burden to the surrounding brain parenchyma while increasing the treatment dose on the lesion [18,19]. Minimally invasive procedures such as laser interstitial thermal therapy (LITT), pain management, or sporadic pharmacological treatment of epilepsy may also hold up as an alternative to surgery. These conservative strategies are particularly well-suited for cases where the DNET exhibits a distinctly indolent, non-aggressive clinical course but still provokes direct, localized pain syndromes. In such scenarios, prioritizing targeted symptom control over radical resection can effectively preserve the patient’s quality of life while mitigating the inherent risks associated with extensive operative interventions. Radiotherapy or radiosurgery was not considered in our case since it is still not the standard of care in dysembryoplastic neuroepithelial tumors.

Given the insidious nature of spinal involvement, obtaining a spinal MRI is highly recommended for specific DNET patients—particularly older adults with atypical presentations, or those facing intracranial recurrence following a subtotal resection—to facilitate the early detection of multifocal lesions or secondary dissemination [3]. Because navigating these diagnostic and therapeutic complexities can be highly challenging, a robust multidisciplinary approach integrating neurosurgery, neuroradiology, and neuropathology is essential. To further optimize the care of this rare entity, future research must prioritize molecular profiling to elucidate the genetic underpinnings of multifocal DNETs and identify potential therapeutic targets. Ultimately, establishing international case registries will be crucial for aggregating clinical data on a global scale, thereby improving our overarching understanding of DNET epidemiology, pathogenesis, and long-term patient-tailored management strategies.

This study highlights the unique challenges of disseminated intracranial and spinal DNETs, advocating for comprehensive neuroimaging, precise surgical techniques, and collaborative care to optimize outcomes in this rare multifocal presentation.

## 6. Limitations

The present study had several limitations that need to be acknowledged. The retrospective design and reliance on published cases introduce selection and publication bias, favoring diagnostically confirmed or unusual presentations. The rarity of such a presentation comes with the downside of a rather modest sample size included in the systematic review, which limits the statistical power of any comparative analysis and the ability to draw definitive conclusions about prevalence or causality. The scarcity of disseminated DNETs hampers our ability to make conclusions regarding the ideal management and we should therefore maintain a patient-centered approach with individualized care in each case. Furthermore, the data were extracted from heterogeneous sources with varying levels of detail, leading to inevitable inconsistencies in reporting and potential gaps in key clinical or histopathological variables. Only English-language reports were included, potentially excluding other relevant cases.

## 7. Conclusions

This case of a 47-year-old woman with disseminated intracranial and spinal DNET, successfully managed with surgical resection, highlights a rare multifocal presentation. Rarity of this case manifests in the patient’s age, atypical clinical features (including false lateralization) and, most importantly, the disseminated lesions. A systematic review of five cases underscores the diagnostic challenges and variable outcomes of intracranial and spinal tumors. The disseminated intracranial and spinal DNETs show distinct clinical features compared to solitary intracranial manifestations, the most outstanding being the low incidence of epilepsy, noted solely in our present case. Given the indolent nature of DNETs, minimally invasive procedures such as laser interstitial thermal therapy (LITT), pain management, or sporadic pharmacological treatment of epilepsy may hold up as an alternative to surgery.

We acknowledge that institutional and financial constraints in this case limited full adherence to the ideal diagnostic and surgical gold standard. For optimal management of spinal DNETs, a comprehensive protocol—including whole-spine MRI, CSF cytology, and molecular profiling—should be prioritized, similarly to ependymoma treatment. Furthermore, interdisciplinary consensus is essential to evaluate the necessity of fusion surgery for stability and potentially safer techniques like the subtemporal approach.

While our patient remains recurrence-free following multidisciplinary review, emphasizing these ‘gold standard’ steps provides a necessary roadmap for treating such rare neuro-oncological challenges, where attaining an ideal therapeutic trajectory without specific guidelines remains a formidable task.

## Figures and Tables

**Table 1:** Clinical features and course of hospitalization—6 cases of concurrent intracranial and spinal dysembryoplastic neuroepithelial tumors.

C.N.	Age/Sex	Initial Diagnosis	Primary Tumor	Metastasis	Symptoms on Onset	New/Postoperative Symptoms	Surgery	Biopsy	Treatment	WNV	Fatal
1	29/F	Intraventricular astrocytoma	Entire ventricles	Cauda equina, S1 nerve roots	Decreased visual acuity, hand tremors, gait disturbance, drowsy mental status, diplopia	Lumbar pain, motor weakness in both legs	-	Right LV lesion (temporal horn)	Radiation therapy to the sacral region	-	-
2	9/M	Differentiation between ODG, CNC, SEGA, or DNET	Third ventricle	Cervicothoracal and lumbar spinal cord	Headache, back pain, nausea, bilateral papilledema	Stability	Anterior interhemispheric transcallosal approach; subtotal resection	Spinal lesion	No radio- or chemotherapy	-	-
3	36/M	Leptomeningeal spread from known thoracic lesion	T2-T3 thoracic spinal cord	Filum terminale, lumbar and sacral nerve roots + intracranial *	Migraine, blurred vision, general weakness, numbness, urinary incontinence, vomiting, weakness in left limb, left positive Babinski sign	Headaches, enuresis, decreased visual acuity, tonsillar herniation, neck, lower back and left thigh pain, worsening of lower extremity weakness, showed WNV activity, ultimately quadriparetic	Lumbar laminectomy	Lumbar subdural lesion	Hospice care	7 months prior	+
4	22/-	Unknown lesions of third ventricles	Third ventricle (bottom and lateral walls)	Anterior horns of lateral ventricles, cerebellar vermis, cervical and lumbar spinal	Visual discomfort, cloudy vision, headaches, papilledema	Partial regression of optic disc congestion	Septosomy	The tumor in third and lateral ventricles	Craniospinal irradiation, boost radiotherapy to chiasmatic-sellar tumor, LV and lumbar spinal cord	-	-
5	35/M	Effect of previous WNV infection	Lumbar spinal cord	Cerebellum, meninges	Hydrocephalus	Quadriplegia, chronic spinal cord inflammation	-	Lumbar, cerebellar lesion, meninges	Steroids, hospice care	8 months prior	+
6	47/F	Cerebral and supratentorial neoplasm of unknown origin	Surrounding right hippocampus; exophytic growth into the subarachnoid cisterns at the skull base	Intradural L5-S1 lesion	Dizziness, heavy head sensation	Lumbosacral pain; periodically radiating to the left lower limb through the buttock, laterally in the thigh, calf up to the left lateral malleolus (pain worsens while standing)	Right side supraorbital craniotomy; total resection + L5 laminectomy with intraoperative neuromonitoring, percutaneous facet joint denervation (L3/L4 and L4/L5)	-	Pain management by denervation	-	-

C.N. = case number, ODG = oligodendroglioma, CNC = central neurocytoma, SEGA = subependymal giant cell astrocytoma, LV = lateral ventricle. * bilateral cerebelli, brainstem, right frontal and temporal lobes.

## Data Availability

No new data were created or analyzed in this study.
